# Characterization of gut microbiome composition in Iranian patients with nonalcoholic fatty liver disease and nonalcoholic steatohepatitis

**DOI:** 10.1038/s41598-023-47905-z

**Published:** 2023-11-23

**Authors:** Sara Abdollahiyan, Ali Nabavi-Rad, Shahrbanoo Keshavarz Azizi Raftar, Magali Monnoye, Naghmeh Salarieh, Azam Farahanie, Hamid Asadzadeh Aghdaei, Mohammad Reza Zali, Behzad Hatami, Philippe Gérard, Abbas Yadegar

**Affiliations:** 1 Foodborne and Waterborne Diseases Research Center, Research Institute for Gastroenterology and Liver Diseases, Shahid Beheshti University of Medical Sciences, Tehran, Iran; 2 Micalis Institute, INRAE, AgroParisTech, Paris-Saclay University, Jouy-en-Josas, France; 3 Gastroenterology and Liver Diseases Research Center, Research Institute for Gastroenterology and Liver Diseases, Shahid Beheshti University of Medical Sciences, Tehran, Iran; 4 Basic and Molecular Epidemiology of Gastrointestinal Disorders Research Center, Research Institute for Gastroenterology and Liver Diseases, Shahid Beheshti University of Medical Sciences, Tehran, Iran

**Keywords:** Hepatology, Microbial communities

## Abstract

Gut microbiota dysbiosis is intimately associated with development of non-alcoholic fatty liver disease (NAFLD) and nonalcoholic steatohepatitis (NASH). Nevertheless, the gut microbial community during the course of NAFLD and NASH is yet to be comprehensively profiled. This study evaluated alterations in fecal microbiota composition in Iranian patients with NAFLD and NASH compared with healthy individuals. This cross-sectional study enrolled 15 NAFLD, 15 NASH patients, and 20 healthy controls, and their clinical parameters were examined. The taxonomic composition of the fecal microbiota was determined by sequencing the V3-V4 region of 16S rRNA genes of stool samples. Compared to the healthy controls, NAFLD and NASH patients presented reduced bacterial diversity and richness. We noticed a reduction in the relative abundance of Bacteroidota and a promotion in the relative abundance of Proteobacteria in NAFLD and NASH patients. L-histidine degradation I pathway, pyridoxal 5'-phosphate biosynthesis I pathway, and superpathway of pyridoxal 5'-phosphate biosynthesis and salvage were more abundant in NAFLD patients than in healthy individuals. This study examined fecal microbiota dysbiosis in NAFLD and NASH patients and presented consistent results to European countries. These condition- and ethnicity-specific data could provide different diagnostic signatures and therapeutic targets.

## Introduction

Non-alcoholic fatty liver disease (NAFLD) has been known as the most common chronic liver disease, frequently resulting in morbidity and mortality worldwide^1^. NAFLD initiates with simple hepatic steatosis and could progress to nonalcoholic steatohepatitis (NASH), liver fibrosis, cirrhosis, and eventually hepatocellular carcinoma^2,3^. According to the iteration of the Global Burden of Disease (GBD) in 2017, NASH was the primary cause of cirrhosis among Iranian patients, with a prevalence of approximately 18 million subjects and an age-standardized prevalence rate of 20,500 per 100,000. Moreover, cirrhosis and other chronic hepatic disorders were reported to account for 1.42% of total deaths in that year^4^.

Fatty liver was defined based on the clinic pathological term in which triglycerides are augmented in hepatocytes^5,6^. The “first hit”, which results in NAFLD promotion, refers to an increased level of free fatty acids (FFAs) in adipocytes and a reduction in the oxidation of FFAs in the liver, leading to excessive accumulation of fat in hepatic cells. The “second hit” refers to the secretion of inflammatory cytokines and the expansion of oxidative stress, which results in persistent damage to the liver tissue^7^. However, several other parameters such as the intricate interaction of genetic^8,9^, environmental, dietary, and metabolic factors as well as the gut microbiome, can affect the incidence of NAFLD and NASH, which is referred to as the multiple-hit hypothesis^10–12^. The human gastrointestinal tract harbors a complex community of resident microbes that is mainly composed of commensal bacteria, as well as fungi, viruses, archaea, and Protista, whose significant involvement in the gut ecosystem has only recently begun to be acknowledged^13^. The gut microbiota plays significant roles in physiological and pathological conditions of human health, taking part far beyond digestion and participating in the regulating of metabolic pathways, host immune response, angiogenesis, circulation, and nervous system activity^14,15^.

Recently, gut microbiota has been suggested as a main contributor to NAFLD development^16,17^. Gut microbiota not only does participate in the overall well-being of the hosts but also plays a critical role in the maintenance of liver homeostasis since the liver is the first organ to drain the gut via the portal vein^18^. Therefore, the liver is more readily exposed to gut microbiota or associated digested microbial products, and this interconnection is called the gut-liver axis or the liver-microbiome axis^19^. Gut dysbiosis potentially induce NAFLD development by disrupting the gut-liver axis, ultimately increasing gut permeability and unrestrained translocation of microbial metabolites and by-products into the hepatic tissue^20^.

To date, multiple studies have reported gut dysbiosis and alterations in the intestinal microbial profile in NAFLD and NASH patients^21–24^. However, there have been discrepancies among these studies, which might have stemmed from the differences in clinical and demographic features of the target population, heterogeneity in terms of the microbiota analysis techniques as well as diet and lifestyle in different geographical regions. Moreover, no study has yet examined changes in the fecal microbiota composition of Iranian patients with NAFLD and NASH. Therefore, the present study aimed to assess alterations in fecal microbiota in a population of NAFLD and NASH patients along with healthy controls from Iran.

## Material and methods

### Study design, setting, and participants

This was a single-center, cross-sectional research conducted at the Research Institute of Gastroenterology and Liver Disease (RIGLD), Shahid Beheshti University of Medical Sciences, Tehran, Iran. Between July 2020 to February 2022, 15 NAFLD, 15 NASH patients, and 20 healthy subjects who were referred to the Liver Clinic of RIGLD were recruited into this study. NAFLD diagnosis was made by transient elastography (FibroScan, Echosence, France) and described as presenting a controlled attenuation parameter (CAP) score above 260 dB/m. NASH was described as CAP > 260 dB/m and serum ALT level > 45 IU/L. Individuals with CAP < 260 dB/m and normal ALT levels were selected as the healthy group^25–28^.

All individuals fulfilled a standardized questionnaire consisting of demographics, antibiotic and medication history, comorbidities, underlying medical condition, and clinical symptoms. All subjects were in the BMI range of 17 < BMI < 35 kg/m^2^. Exclusion criteria were individuals with any of the following conditions: hepatitis and metabolic syndromes (diabetes mellitus and high serum cholesterol), taking antibiotics or probiotics within the preceding 3 months, infection with hepatitis B or C virus, history of excessive alcohol consumption (40 g per week) within the past 12 months, autoimmune disorders, advanced liver disease, or decompensated cirrhosis, and individuals who smoke^29^. This study was approved by the Ethical Review Committee of RIGLD, Shahid Beheshti University of Medical Sciences, Tehran, Iran (Project No. IR.SBMU.RLGLD.REC.1399.019). Written informed consent was obtained from all eligible subjects or their legal representative prior to enrollment in this study. All methods were conducted in accordance with the relevant guidelines and regulations.

### Fecal samples collection

A total of 50 stool samples from the study population were collected and immediately transferred to the microbiology laboratory of RIGLD. The samples were homogenized by agitation with a vortex and aliquoted within 2 h of defecation. The aliquots were then instantly frozen and stored at − 80 °C in screw capped cryovial tubes until used for DNA extraction.

### DNA extraction

Total genomic DNA extraction from fecal samples was performed using the QIAamp Fast DNA Stool Mini Kit (Qiagen Retsch GmbH, Hannover, Germany) following the manufacturer’s protocol with minor modifications. DNA concentration and purity were evaluated by NanoDrop ND-2000 Spectrophotometer (NanoDrop products, Wilmington, DE, USA). DNA extracts were kept at − 20 °C until microbiota analysis^30^.

### Fecal microbial community analysis

The V3-V4 region of the 16S rRNA genes was amplified using Phanta Max Super-Fidelity DNA Polymerase (CliniSciences, Nanterre, France) and primers V3F: 5′-TACGGRAGGCAGCAG-3′ and V4R: 5′-ATCTTACCAGGGTATCTAATCCT-3′ as previously described^31^. The purified amplicons were sequenced using Miseq sequencing technology (Illumina) at the @BRIDGe sequencing facility (GABI, INRAE, AgroParisTech, Paris-Saclay University, Jouy-en-Josas, France). Paired-end reads obtained from MiSeq sequencing were analyzed using the Galaxy-supported FROGS (Find, Rapidly, OTUs (Operational Taxonomic Units) with Galaxy Solution) pipeline^32,33^. For preprocessing, sequences shorter than 410 bp and longer than 480 bp were excluded. Clustering and chimera removal steps were performed in accordance with the FROGS guidelines. Assignation was performed using SILVA 16S V138. Low-abundance OTUs (< 0.005% of the total OTUs) were eliminated before analysis. Thereafter, 16S sequencing data were analyzed using the Phyloseq, and ggplot2 R packages in addition to custom scripts as described previously^33^. Raw, unrarefied OTU counts were used to produce relative abundance graphs and to find taxa with significantly different abundances. Samples were rarefied to even sampling depths (sequencing depth: 5374) prior to computing to alpha- and beta-diversity indices.

PICRUSt2 was used under default settings to predict a profile of putative microbial functions (via metagenome prediction) from the 16S rRNA sequences. Representative sequences were analyzed using PICRUSt2 and classified against the MetaCyc database of metabolic pathways and enzymes^34^. The datasets produced in the present study are available in the following database: Recherche Data Gouv under the accession number: https://doi.org/10.57745/MYY4DU.

### Statistical methods

The clinical data are expressed as mean ± SD (standard deviation). Statistical differences were assessed using one-way ANOVA and Tukey post-hoc test, performed by GraphPad Prism 5 software version 5.04 (GraphPad Software, Inc., San Diego, CA, USA). For microbiota analysis, we used an ANOVA followed by a Tukey post-hoc test for pairwise comparisons of the alpha-diversity indices. Principal Coordinate Analysis was performed and a permutational multivariate ANOVA test was done on the Jaccard, Bray–Curtis, Unifrac, and weighted Unifrac matrices using 9999 random permutations. Relative abundances at the phylum and family levels were compared using the non-parametric Kruskal–Wallis test and Dunn post-hoc test using the Benjamini–Hochberg procedure. Differential abundance of taxa (OTUs) was tested using negative binomial model implemented in DESeq2^35^ and *P* values corrected with False Discovery Rate (FDR) procedure. Differential abundance of PICRUSt2-inferred pathways was identified using DESeq2.

### Ethics approval and consent to participate

Informed consent was obtained from all individual participants included in the study. All procedures performed were following the ethical standards retrieved from the Institutional Ethical Review Committee of the Research Institute for Gastroenterology and Liver Diseases at Shahid Beheshti University of Medical Sciences (Project No. IR.SBMU.RLGLD.REC.1399.019).

## Results

### Characteristics of study population

We enrolled a total of 15 NAFLD, 15 NASH, and 20 healthy subjects in this study. Demographic data and clinical characteristics of the study population are presented in Table 1. Alanine transaminase (ALT) and aspartate transaminase (AST) were substantially higher in NAFLD (*P* = 0.02) and NASH (*P* = 0.02) patients, compared to the healthy group. Direct bilirubin was also markedly higher in NAFLD (*P* = 0.006) and NASH (*P* = 0.03) patients. Furthermore, in comparison to the healthy controls, patients with NAFLD had a notably elevated level of creatine (*P* = 0.04) while patients with NASH had a substantially elevated level of BUN (*P* = 0.008).

**Table 1:** Demographic data and clinical characteristics of all patients and healthy control enrolled in this study.

Variable	NAFLD (n = 15)	NASH (n = 15)	Healthy control (n = 30)	*P* value NAFLD vs. HC	*P* value NASH vs. HC
Age, y range	46.20 ± 12.049 (21–64)	45.67 ± 14.720 (22–78)	33.35 ± 8.375 (23–59)	0.1	0.3
Gender, n (%) male female	7 (46.7) 8 (53.3)	8 (53.3) 7 (46.7)	6 (30.0) 14 (70.0)	0.3	0.1
BMI (kg/m^2^) range	30.88 ± 4.023 (24.5–39.1)	31.88 ± 5.208 (24.4–41.3)	23.28 ± 3.914 (17.5–30.8)	0.4	0.3
ALT (U/L) range	34.20 ± 13.208 (12–55)	73.73 ± 31.093 (34–154)	13.15 ± 1.663 (11–16)	**0.02**	**0.02**
AST (U/L) range	27.87 ± 10.941 (13–49)	67.13 ± 65.110 (28–266)	15.40 ± 1.984 (12–19)	**0.02**	**0.02**
ALP (U/L) range	159.47 ± 55.150 (19–293)	170.0 ± 66.722 (50–336)	88.55 ± 15.466 (72–142)	0.2	0.1
Total bilirubin (mg/dL) range	1.04 ± 0.286 (0.6–1.5)	0.68 ± 0.218 (0.30–1.10)	0.42 ± 0.167 (0.28–1.00)	0.1	0.1
Direct bilirubin (mg/dL) range	0.33 ± 0.132 (0.1–0.5)	0.26 ± 0.134 (0.10–0.60)	0.12 ± 0.026 (0.10–0.19)	**0.006**	**0.03**
Albumin (g/dL) range	4.68 ± 0.405 (3.8–5.3)	4.47 ± 0.502 (3.4–5.1)	4.34 ± 0.214 (4.0–4.7)	0.2	0.3
INR range	1.00 ± 0.027 (0.97–1.00)	1.01 ± 0.036 (1.00–1.14)	1.01 ± 0.046 (1.00–1.21)	0.2	0.3
BUN (mg/dL) range	13.08 ± 3.206 (8.00–20.55)	14.20 ± 3.337 (8.00–19.00)	11.05 ± 1.468 (9.00–14.00)	0.07	**0.008**
Creatine (mg/dL) range	0.94 ± 0.163 (0.70–1.27)	0.99 ± 0.229 (0.70–1.60)	0.71 ± 0.144 (0.50–0.97)	**0.04**	0.07
TG (mg/dL) range	136.07 ± 55.787 (51–238)	152.08 ± 63.876 (53–298)	77.50 ± 16.000 (57–124)	0.2	0.2
Cholesterol (mg/dL) range	186.80 ± 33.849 (143–252)	168.90 ± 64.523 (112–271)	140.90 ± 28.305 (100–212)	0.5	0.5
FBS (mg/dL) range	100.93 ± 9.246 (85–119)	99.67 ± 17.646 (78–156)	94.35 ± 7.909 (81–115)	0.1	0.2
RBC (10^6^/μL) range	5.03 ± 0.479 (4.50–5.92)	5.30 ± 0.370 (4.51–5.80)	4.43 ± 0.559 (3.80–5.50)	0.2	0.1
WBC (10^3^/μL) range	6.57 ± 1.352 (4.70–9.84)	7.12 ± 2.182 (3.95–11.50)	5.09 ± 0.698 (4.00–6.64)	0.4	0.4
PLT (10^3^/mL) range	241.47 ± 42.571 (177–335)	276.00 ± 147.774 (147–760)	285.50 ± 55.099 (174–379)	0.3	0.5
HB (g/dL) range	14.56 ± 1.459 (12.0–16.6)	14.22 ± 1.120 (12.3–16.3)	12.65 ± 0.952 (11.2–14.3)	0.2	0.2
HCT (%) range	42.86 ± 5.351 (32.2–50.2)	41.78 ± 3.448 (36.5–47.6)	36.35 ± 2.872 (32.1–41.9)	0.3	0.4

Significant values are in bold.

*P* < 0.05; (mean ± SD).

*SD* standard deviation, *NAFLD* Non-alcoholic fatty liver disease, *NASH* Nonalcoholic steatohepatitis, *BMI* Body mass index, *ALT* Alanine aminotransferase, *AST* Aspartate transaminase, *INR* International normalized ratio, *BUN* Blood urea nitrogen, *TG* Triglyceride, *FBS* Fasting blood sugar, *RBC* Red blood cell, *WBC* White blood cell, *PLT* Platelets *HB*, Hemoglobin, *HCT* Hematocrit.

### Dysbiosis of microbiome diversity in NAFLD and NASH patients

**Figure 1:**
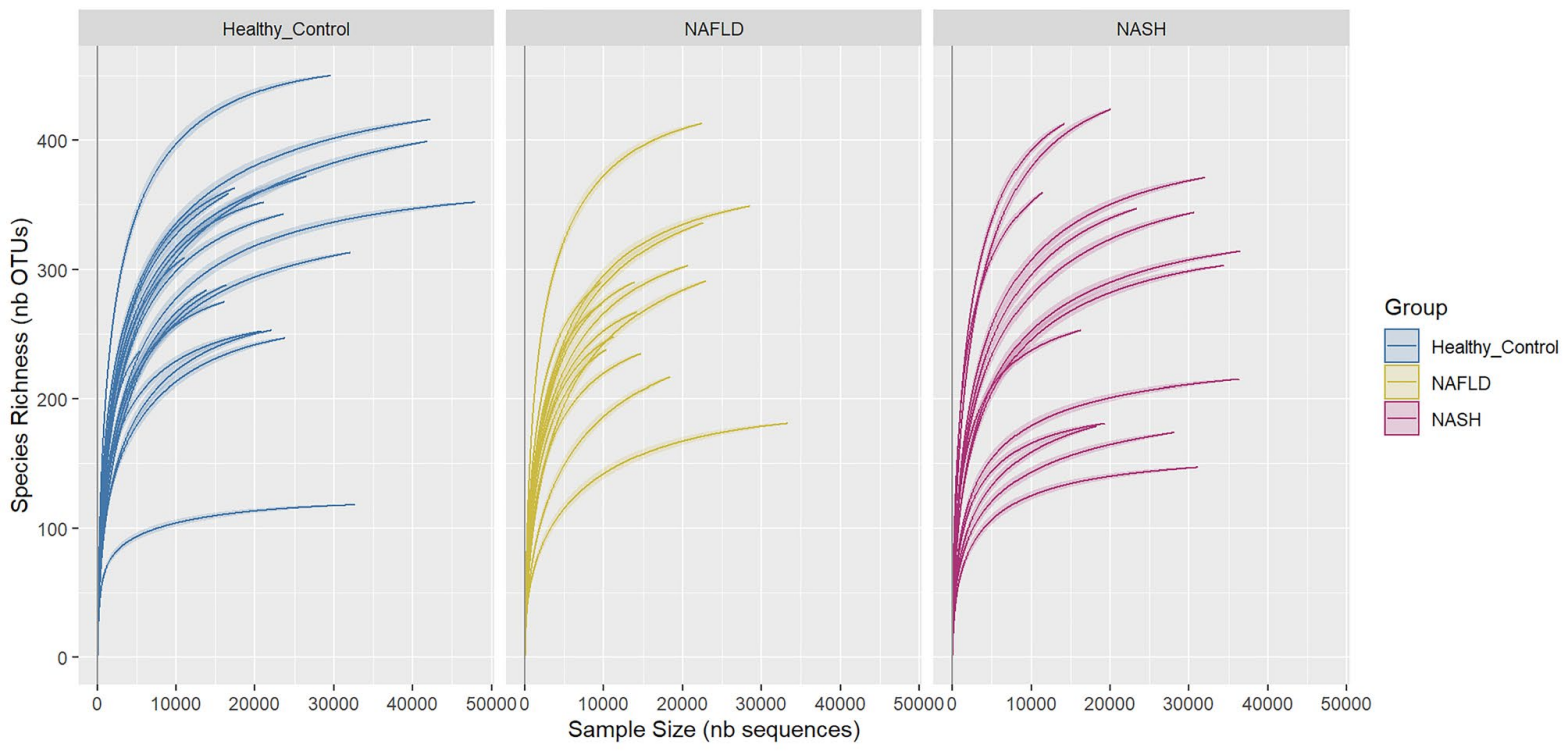
Rarefaction curves of all 16S rRNA amplicon for fecal samples in each study group based on Miseq sequencing technology (Illumina).

A total of 1,067,331 reads were sequenced and passed the quality filters. These reads were further assigned into 1,019 OTUs, 100 and 89.0% of which were successfully assigned at the phylum and genus levels, respectively. The median read count per sample was 22,705, and rarefaction curve analysis showed that the majority of samples reach saturation, indicating sufficient sequencing depth to capture microbiome diversity (Fig. 1).

**Figure 2:**
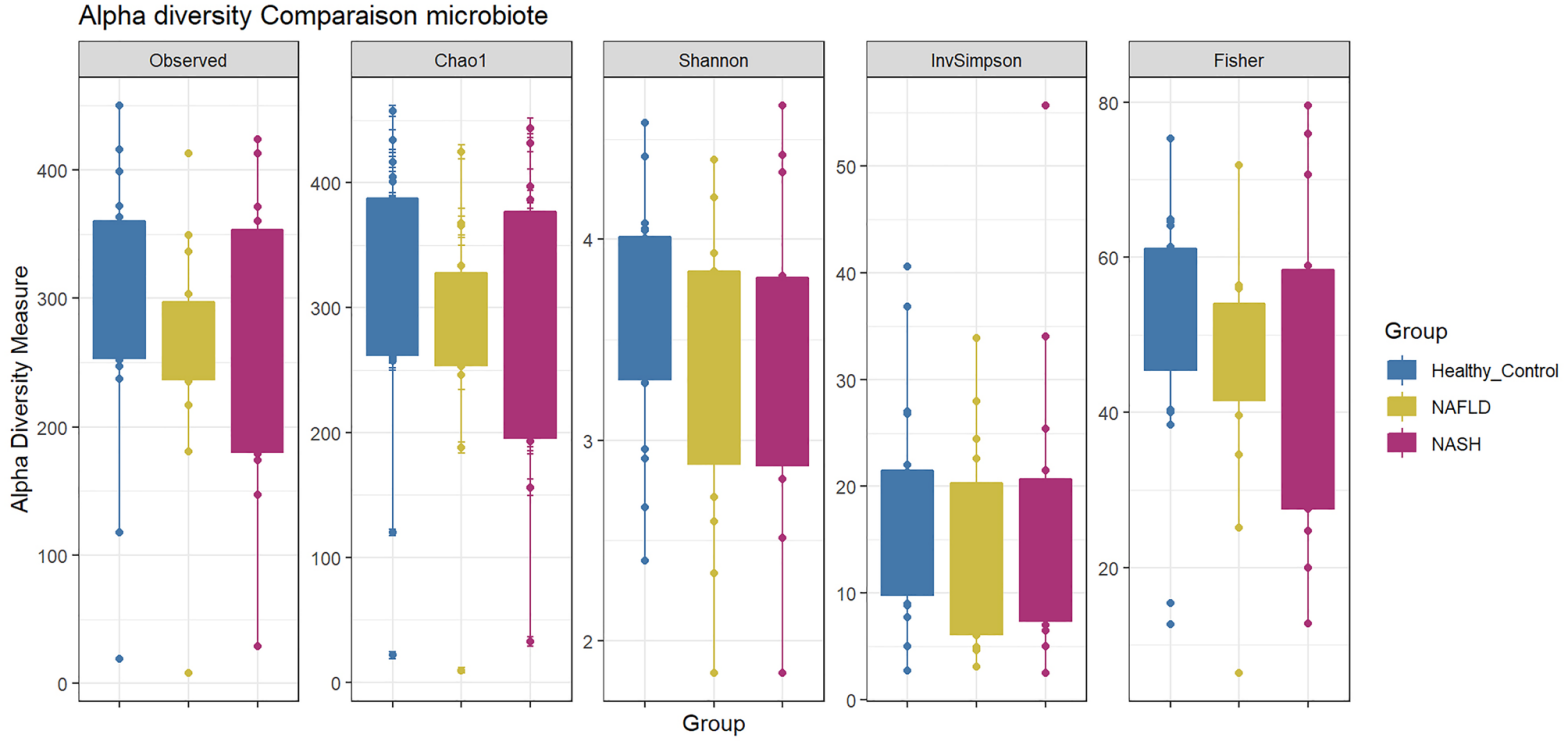
Alpha diversity measure using observed, Chao1, Shannon, InvSimpson and Fisher indices. The significance of differences between diversities were evaluated by Tukey post-hoc test statistical analysis.

**Figure 3:**
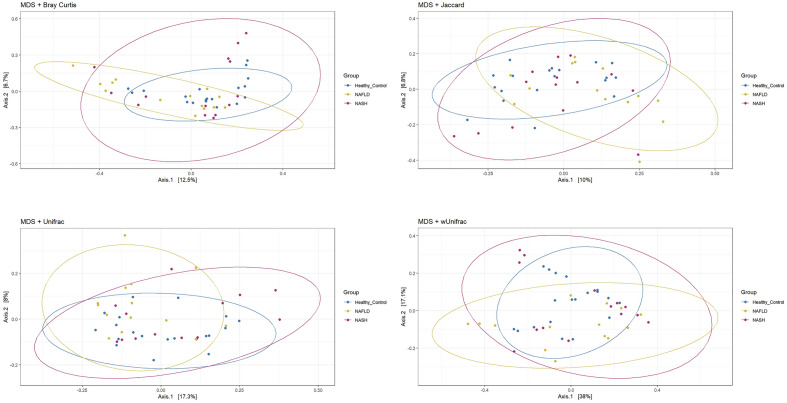
PCA plot based on the Bray–Curtis dissimilarity, Jaccard distance, UniFrac distance, and weighted UniFrac (wUnifrac) distance between samples. Samples from healthy, NAFLD, and NASH patients are not clearly distinct from each other, indicating delicate differences in microbiome structures.

Alpha diversities of fecal microbiota were measured using observed, Chao1, Shannon, InvSimpson, and Fisher indices (Fig. 2). Although alpha diversity alteration was inconspicuous, all diversity indices present a reduced trend in NASH and more notably in NAFLD patients. All diversity indices followed similar trends indicating that the number of species, their abundance and their distribution were not different between groups. Furthermore, to quantify the difference in the fecal microbiota composition, beta diversities were measured using both non-phylogenetic (Bray–Curtis dissimilarity, Jaccard distance) and phylogenetic (UniFrac distance) methods (Fig. 3). Interestingly, while the different plots did not clearly separated samples from healthy, NAFLD, and NASH patients, statistical analysis revealed significant differences using Jaccard (*P* = 0.026) and Unifrac (*P* = 0.038) indices whereas non significance was obtained with Bray–Curtis and Weighted Unifrac. This indicates subtle differences between microbiota communities based on presence/absence of taxa more than on abundance.

### Characteristics of the fecal microbiota in NAFLD and NASH patients

**Figure 4:**
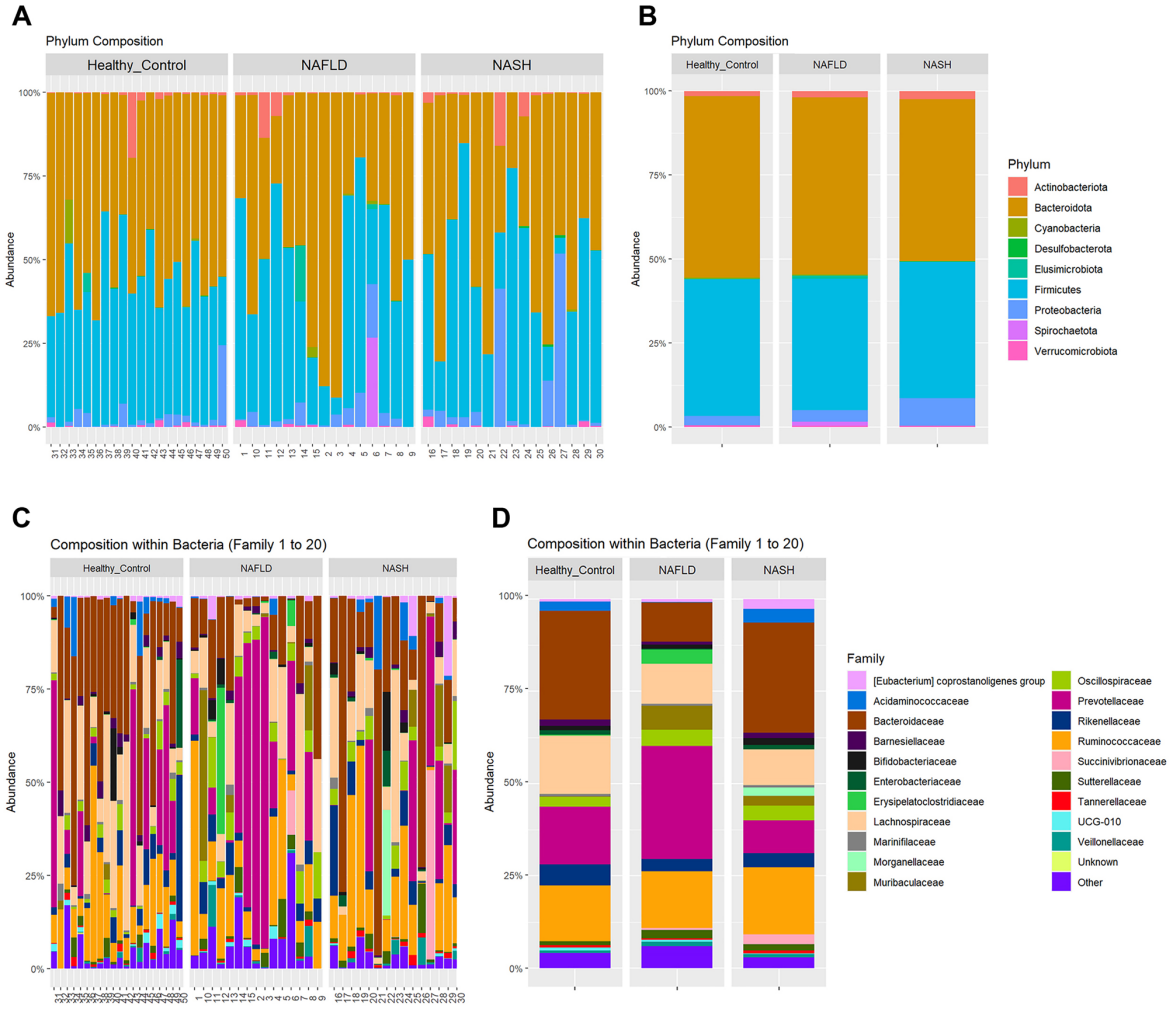
The relative percentage and alteration of the gut microbiota in stool samples of healthy controls, NAFLD, and NASH patients. (**A**) Phylum-level composition of the gut microbiota in each individual. (**B**) Phylum-level composition of the gut microbiota in each group. (**C**) Family-level composition of the gut microbiota in each individual. (**D**) Family-level composition of the gut microbiota in each group. Each color represents a type of microbiota analyzed in this study.

We assessed the relative abundance of the fecal microbiota at the phylum and family levels among NAFLD and NASH patients (Fig. 4). At the phylum level, the fecal microbiota of NAFLD patients is dominated by Bacteroidota (48.2%), Firmicutes (42.4%), Proteobacteria (3.8%), and Elusimicrobiota (1.2%) while fecal microbiota of NASH patients is dominated by Bacteroidota (48.3%), Firmicutes (40.4%), Proteobacteria (8.4%), and Actinobacteriota (2.0%). Compared with healthy individuals, there was a slight decrease in the relative abundance of Bacteroidota and a minor increase in the relative abundance of Proteobacteria in NAFLD and NASH patients. At the family level, however, NAFLD patients presented a remarkable reduction in the relative abundance of Bacteroidaceae (*P* = 0.025), Marinifilaceae (*P* = 0.028), and Pasteurellaceae (*P* = 0.043).

**Figure 5:**
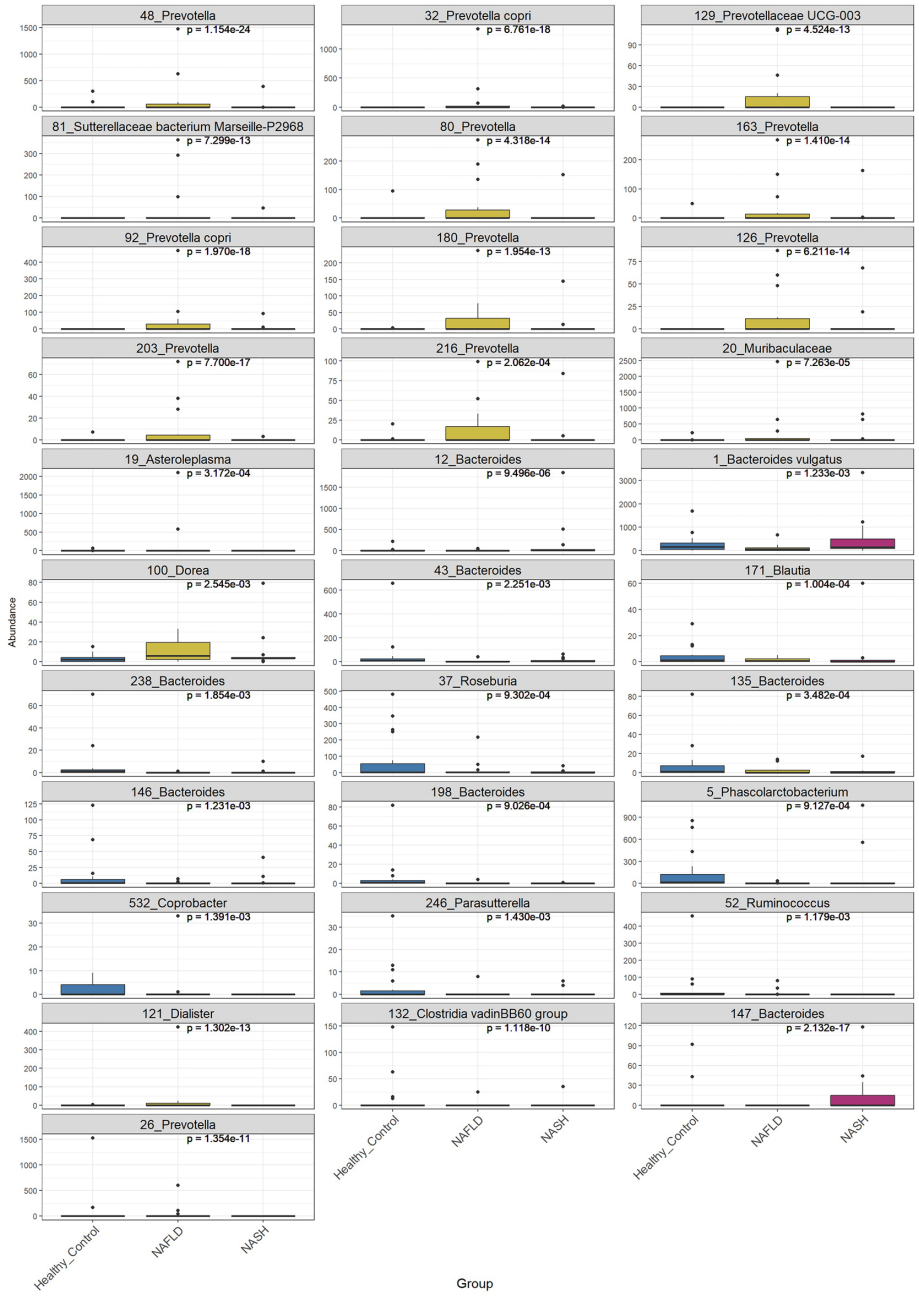
Boxplots of OTUs for which the abundance was significantly different between healthy controls, NAFLD, and NASH patients. Control vs NAFLD (9 up and 9 down), control vs NASH (2 up and 2 down), and NAFLD vs NASH (6 up and 1 down). (*P < 0.05; **P < 0.01; ***P < 0.001; ****P < 0.0001).

To further substantiate bacterial OTUs correlated with NAFLD and NASH, the pairwise comparison of differential abundance was evaluated using the Wald test of DESeq2. All pairwise comparisons (control vs NAFLD; control vs NASH; NAFLD vs NASH; adjusted *P* < 0.05) presented a total of 29 differentially abundant distinct OTUs (Fig. 5). These OTUs were made up of 18 (control vs NAFLD, 9 up and 9 down), 4 (control vs NASH, 2 up and 2 down), and 7 (NAFLD vs NASH, 6 up and 1 down) unique OTUs. Interestingly, several OTUs more abundant in healthy controls belong to known butyrate-producing genera (*Blautia*, *Roseburia*, *Phascolarctobacterium*) suggesting that a deficit in butyrate production may be a characteristic of the microbiota of NAFLD/NASH patients. Conversely, several OTUs found decreased in healthy controls have been identified as *Prevotella* species, including *Prevotella copri* which has been previously associated with liver diseases^36,37^.

### Fecal microbiota dysbiosis and serum metabolic parameters

**Figure 6:**
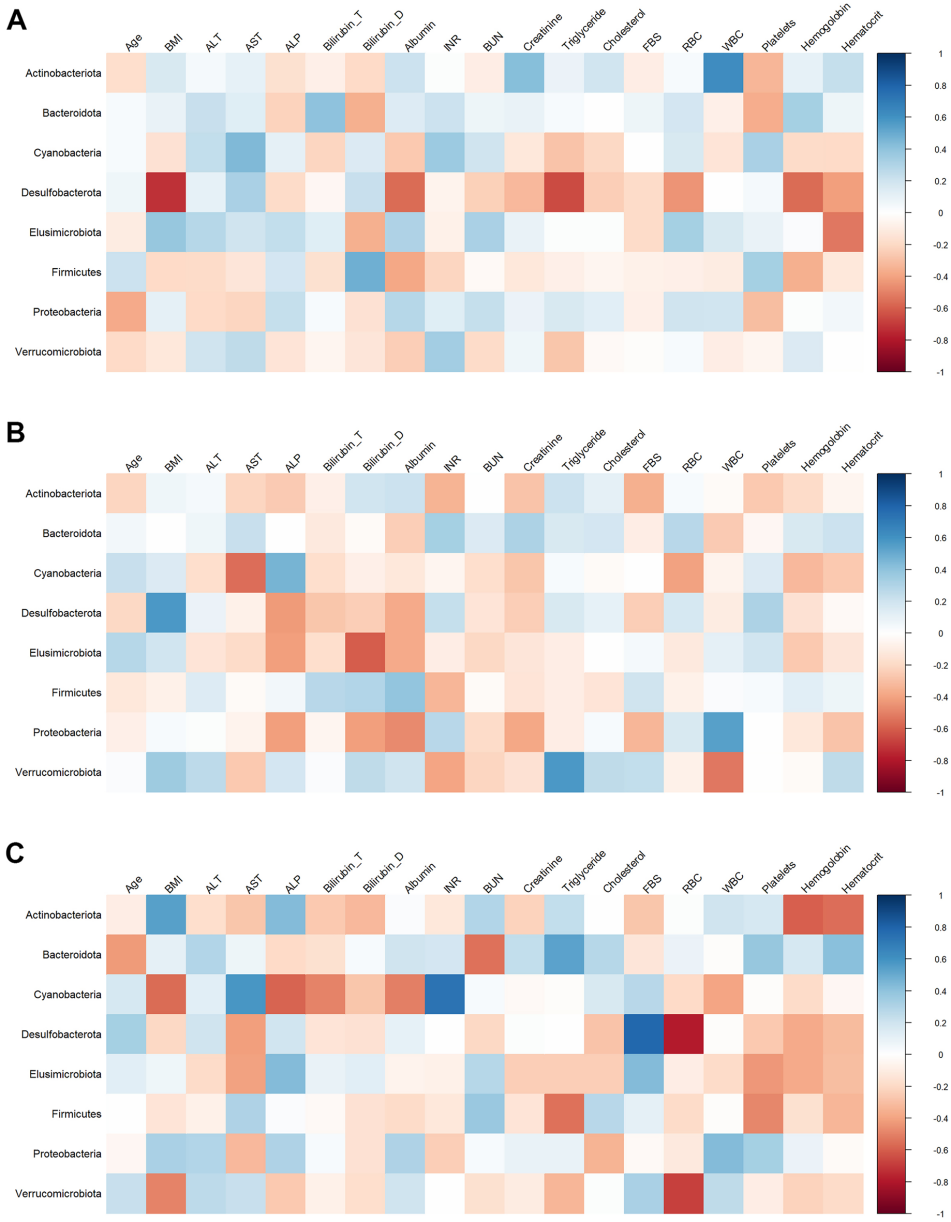
Correlation plot between individuals’ metadata and fecal microbiota composition at the phylum-level, (**A**) healthy controls, (**B**) NAFLD, and (**C**) NASH patients. Spearman correlation presented no statistically significant correlation between the indices. The heatmaps have been generated using the R package corrplot (version 0.92, https://www.rdocumentation.org/packages/corrplot/versions/0.92).

Presuming that the gut microbiota is associated with the pathogenesis and progression of various liver disorders, we calculated the correlations between the fecal microbiota composition and individuals’ demographic data and laboratory characteristics by Spearman correlation (Fig. 6). Although there was no substantial correlation with the indices, we noticed that Marinifilaceae and Pasteurellaceae families, which were impoverished in NAFLD patients, were positively correlated with patients’ BMI (Fig. S1). Furthermore, a higher abundance of Proteobacteria, which includes various pathogenic species, was positively correlated with white blood cell (WBC) in NAFLD and NASH patients.

### Fecal microbiota dysbiosis and metabolic pathways

**Figure 7:**
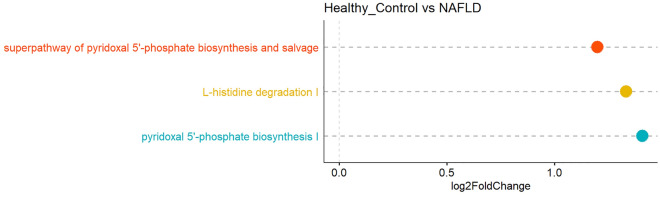
Differentially abundant pathways in the gut microbiome of healthy controls and NAFLD patients using Picrust analysis. There were no substantial differences comparing the NASH group with the control group or the NAFLD group.

To further evaluate the potential contribution of the gut microbiota to the changes in the serum markers, we used PICRUSt2 to predict microbiota functional abundances based on 16S rRNA sequences. Compared to healthy controls, NAFLD patients presented significant enrichment of L-histidine degradation I pathway, pyridoxal 5'-phosphate biosynthesis I pathway, and superpathway of pyridoxal 5'-phosphate biosynthesis and salvage (Fig. 7) while no pathway was found significantly less abundant. On the contrary, no pathways were markedly different in their presumed abundance when comparing NASH patients with control or NAFLD groups, suggesting that these microbial metabolic pathways may be involved in early stages of liver diseases.

## Discussion

In the past decade, studying the biology of the gut-liver axis has led to comprehending fundamental notions about fatty liver diseases. As a result, intestinal microbiome signatures have emerged in a spectrum of liver disorders from NASH and cirrhosis to hepatocellular carcinoma, indicating the key contribution of microbiota and microbiota-derived factors in liver-associated pathologies^38,39^. Therefore, of immense importance is the identification of protective strains, pathobionts, hepatobiliary microbiome, and liver-derived signals during the course of liver diseases^40^. However, the overwhelming majority of studies characterizing the gut microbiota profile using sequencing methods in the context of liver diseases have been conducted in populations from China, North America and Europe whereas populations from the other parts of the globe have been overlooked. To this end, this study, for the first time in Iran, presented the dysbiosis and alterations of the fecal microbiota from Iranian NAFLD and NASH patients and compared it to fecal microbiota from healthy subjects. Such profiling of shifted microbial communities allows the identification of NAFLD and NASH-related alterations in the gut structure and further enables us to discriminate those taxa contributing to NAFLD and NASH pathogenesis from innocent bystanders and protective taxa. Our results, which presented the altered profile of the fecal microbiota in NAFLD or NASH patients are consistent with recent studies implicating that gut microbiome dysbiosis is associated with the risk of NAFLD development and NAFLD severity^22,41^.

Ecological diversities (alpha and beta) presented no significant differences among NAFLD, NASH, and healthy control groups; however, compared to healthy individuals, NAFLD and NASH patients had apparent lower bacterial diversity and richness. This finding is reminiscent of large population cohort studies indicating a significant reduction of overall bacterial diversity and richness in patients with persistent NAFLD^42^.

NAFLD and NASH patients presented a higher proportion of Proteobacteria, which mostly include Gram-negative pathogenic bacterial species. A recent in vivo study reported that an increased abundance of Proteobacteria could be a biomarker for gut-liver axis-directed NAFLD development^43^. Moreover, bacterial species belonging to Proteobacteria have been identified as causative agents of NAFLD^17^. This is mainly due to the fact that the accumulation of Gram-negative bacteria-derived lipopolysaccharide (LPS) in the intestinal mucosa disrupts the integrity of the intestinal epithelial barrier and intestinal vascular barrier, leading to liver inflammation and chronic hepatic damage^44^. Furthermore, LPS-mediated metabolic changes can increase fat consumption and serum levels of FFA and TG, resulting in hepatic FFA deposition, insulin resistance, and NAFLD development^45^. In the present study, Ruminococcaceae, a major SCFA-producing family, was also enriched in NAFLD and NASH patients. The activation of G protein-coupled receptor 43 (GPR43) and subsequent hepatic lipogenesis is the mechanism through which SCFAs (acetate and propionate) contribute to NAFLD development^46^. Conversely, butyrate is considered beneficial for NAFLD and we identified several butyrate producers decreased in NAFLD and NASH patients, highlighting the importance of the type of SCFA produced by the gut microbiota in the context of liver diseases.

Substantially enriched metabolic pathways in NAFLD patients, with respect to the healthy control group, included L-histidine degradation I pathway, pyridoxal 5'-phosphate biosynthesis I pathway, and superpathway of pyridoxal 5'-phosphate biosynthesis and salvage. Driuchina et al.^47^ similarly reported that histidine degradation products N-omega-acetylhistamine and anserine were notably increased in patients with high liver fat. Therefore, histidine degradation was suggested as a potential gut microbiota biomarker of high liver fat. Notably, a positive correlation has been reported between Actinobacteria phylum and L-histidine degradation I pathway in patients with neurodegenerative disorder^48^. This might also be the case in our study as NAFLD patients presented a slightly higher prevalence of Actinobacteria. Regarding pyridoxal 5'-phosphate, however, prior studies demonstrated a lower serum level of pyridoxal 5'-phosphate and a reduction in its biosynthesis pathway in NAFLD patients. Although there is an absence of sufficient documentation to support the role of pyridoxal 5'-phosphate in NAFLD, the potential mechanism suggested for the influence of pyridoxal 5'-phosphate deficiency on NAFLD pathogenesis is the impairment of polyunsaturated fatty acid (PUFA) interconversion^49,50^.

Given the intimate connection between the microbial structure and NAFLD pathogenicity, the dysbiotic characterization of fecal microbiota and metabolome has been suggested as a diagnostic signature for fatty liver diseases. In light of recent developments in machine learning, diagnostic models have facilitated the detection of disease-specific signatures with 80% of overall accuracy^51,52^. Furthermore, machine learning has led to the emergence of classification models to screen NAFLD patients in a general population, which could benefit the patient from early diagnosis^53^. Understanding different mechanistic actions of the gut microbiota that contribute to the pathophysiology of NAFLD and NASH may accelerate the development of more accurate diagnostic models. A holistic comprehension of this cross-talk can further improve microbiome-based therapeutics such as the transplantation of defined microbial consortia. One of the major pillars of microbiome-based therapeutics, fecal microbiota transplantation (FMT) was reported to restore a balanced microbial and metabolic profile in high-fat diet-induced NASH in mouse models. Recently, a clinical trial study also demonstrated that restoring the indigenous composition of the gut microbiota by FMT can decrease fat accumulation in the liver and attenuate fatty liver disease^54^. Furthermore, co-supplementation of microbial product bile acid ursodeoxycholic acid (UDCA) with conventional rosuvastatin/ezetimibe (RSV/EZE) treatment presented a hepatoprotective effect against NAFLD progression in mouse models^55^.

Notwithstanding that this study presented the first microbial profiling of Iranian NAFLD patients, there are some limitations to our study. First, this is a single-center study and a small study population increases the margin of error and makes it challenging to determine ethnicity-specific microbiota. Second, several serum and fecal metabolic parameters were neglected and excluded from our analysis. Third, the metagenomic data from fecal samples were analyzed in the absence of metatranscriptomic and metabolomic data. Therefore, future studies should consider a large-scale multi-ethnic population with a multi-omics approach to better investigate the contribution of microbiota to the pathophysiology and clinical outcomes of fatty liver diseases.

In summary, this study investigated the fecal microbiota structure and serum biomarkers of Iranian NAFLD and NASH patients, as well as healthy individuals. Our findings indicated that dysbiotic characteristics of fecal microbiota contribute to NAFLD and NASH development. The results from the present study and other microbiota profiling studies lay the foundation for a fecal-based diagnostic test and microbiota-based therapeutic approaches.

### Supplementary Information

Supplementary Figure S1.

## Data Availability

The datasets produced in this study are available in the following database: Recherche Data Gouv under the accession number: https://doi.org/10.57745/MYY4DU. The authors confirm all supporting data, code and protocols have been provided within the article or through supplementary data files.
