# Supplementary material for: Characterization of gut microbiome composition in Iranian patients with nonalcoholic fatty liver disease and nonalcoholic steatohepatitis
Source: Sci Rep. 2023 Nov 23;13:20584. doi: 10.1038/s41598-023-47905-z (PMC10667333; doi:10.1038/s41598-023-47905-z)
Supplement: Supplementary file 1 — Supplementary Figure S1. [file 41598_2023_47905_MOESM1_ESM.pdf]

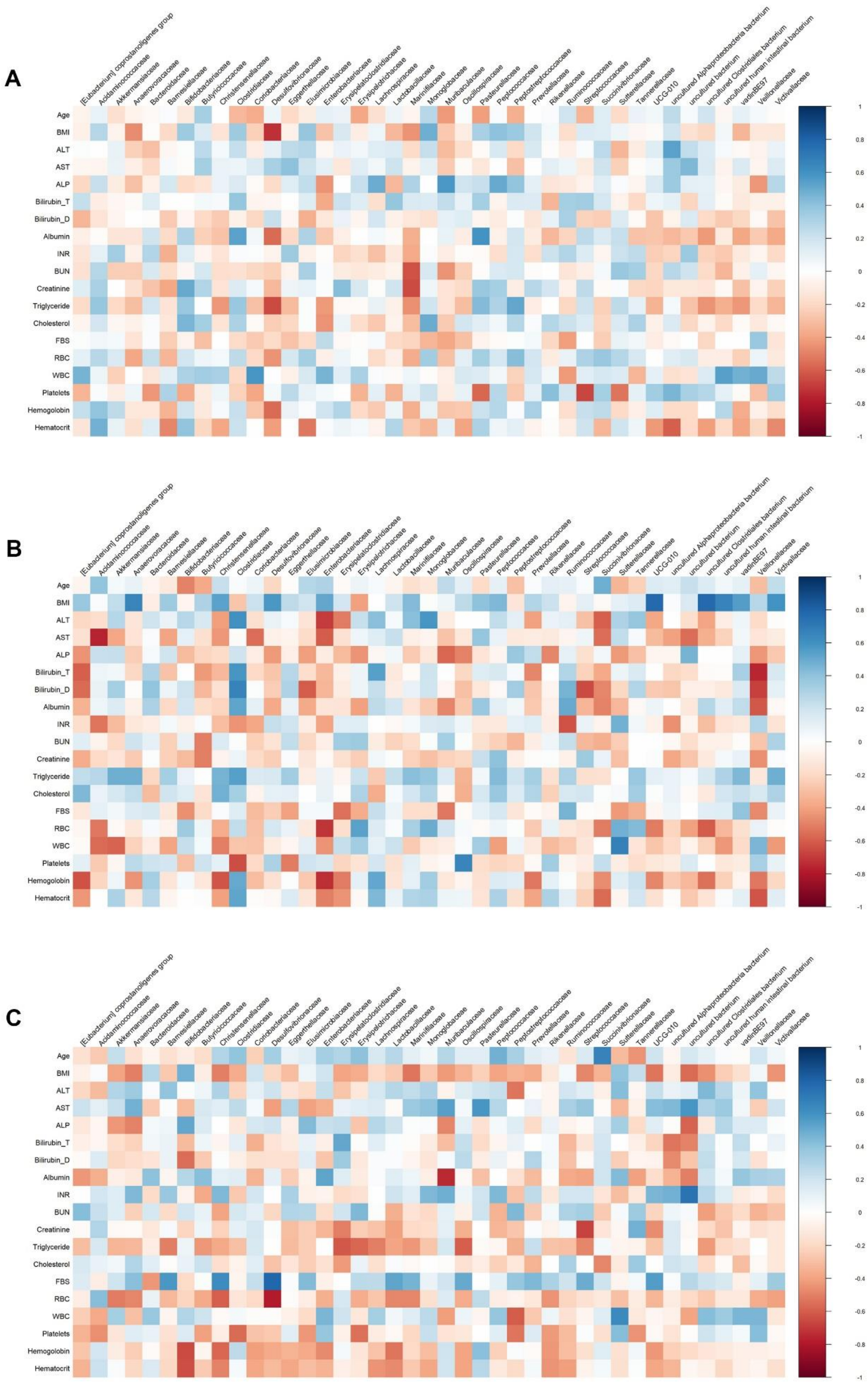
A
[Eubacterium] coprostanoligenes group
Acidaminococcaceae
Akkermansiaceae
Anaerovoracaceae
Bacteroidaceae
Barnesiellaceae
Bifidobacteriaceae
Butyricicoccaceae
Christensenellaceae
Clostridiaceae
Coriobacteriaceae
Desulfovibrionaceae
Eggerthellaceae
Elusimicrobiaceae
Enterobacteriaceae
Erysipelatoclostridiaceae
Erysipelotrichaceae
Lachnospiraceae
Lactobacillaceae
Marinifilaceae
Monoglobaceae
Muribaculaceae
Oscillospiraceae
Pasteurellaceae
Peptococcaceae
Peptostreptococcaceae
Prevotellaceae
Rikenellaceae
Ruminococcaceae
Streptococcaceae
Succinivibrionaceae
Sutterellaceae
Tannerellaceae
UCG-010
uncultured Alphaproteobacteria bacterium
uncultured bacterium
uncultured Clostridiales bacterium
uncultured human intestinal bacterium
vadinBE97
Veillonellaceae
Victivallaceae
Age
BMI
ALT
AST
ALP
Bilirubin_T
Bilirubin_D
Albumin
INR
BUN
Creatinine
Triglyceride
Cholesterol
FBS
RBC
WBC
Platelets
Hemoglobin
Hematocrit
1
0.8
0.6
0.4
0.2
0
-0.2
-0.4
-0.6
-0.8
-1
B
[Eubacterium] coprostanoligenes group
Acidaminococcaceae
Akkermansiaceae
Anaerovoracaceae
Bacteroidaceae
Barnesiellaceae
Bifidobacteriaceae
Butyricicoccaceae
Christensenellaceae
Clostridiaceae
Coriobacteriaceae
Desulfovibrionaceae
Eggerthellaceae
Elusimicrobiaceae
Enterobacteriaceae
Erysipelatoclostridiaceae
Erysipelotrichaceae
Lachnospiraceae
Lactobacillaceae
Marinifilaceae
Monoglobaceae
Muribaculaceae
Oscillospiraceae
Pasteurellaceae
Peptococcaceae
Peptostreptococcaceae
Prevotellaceae
Rikenellaceae
Ruminococcaceae
Streptococcaceae
Succinivibrionaceae
Sutterellaceae
Tannerellaceae
UCG-010
uncultured Alphaproteobacteria bacterium
uncultured bacterium
uncultured Clostridiales bacterium
uncultured human intestinal bacterium
vadinBE97
Veillonellaceae
Victivallaceae
Age
BMI
ALT
AST
ALP
Bilirubin_T
Bilirubin_D
Albumin
INR
BUN
Creatinine
Triglyceride
Cholesterol
FBS
RBC
WBC
Platelets
Hemoglobin
Hematocrit
1
0.8
0.6
0.4
0.2
0
-0.2
-0.4
-0.6
-0.8
-1
C
[Eubacterium] coprostanoligenes group
Acidaminococcaceae
Akkermansiaceae
Anaerovoracaceae
Bacteroidaceae
Barnesiellaceae
Bifidobacteriaceae
Butyricicoccaceae
Christensenellaceae
Clostridiaceae
Coriobacteriaceae
Desulfovibrionaceae
Eggerthellaceae
Elusimicrobiaceae
Enterobacteriaceae
Erysipelatoclostridiaceae
Erysipelotrichaceae
Lachnospiraceae
Lactobacillaceae
Marinifilaceae
Monoglobaceae
Muribaculaceae
Oscillospiraceae
Pasteurellaceae
Peptococcaceae
Peptostreptococcaceae
Prevotellaceae
Rikenellaceae
Ruminococcaceae
Streptococcaceae
Succinivibrionaceae
Sutterellaceae
Tannerellaceae
UCG-010
uncultured Alphaproteobacteria bacterium
uncultured bacterium
uncultured Clostridiales bacterium
uncultured human intestinal bacterium
vadinBE97
Veillonellaceae
Victivallaceae
Age
BMI
ALT
AST
ALP
Bilirubin_T
Bilirubin_D
Albumin
INR
BUN
Creatinine
Triglyceride
Cholesterol
FBS
RBC
WBC
Platelets
Hemoglobin
Hematocrit
1
0.8
0.6
0.4
0.2
0
-0.2
-0.4
-0.6
-0.8
-1

Fig. S1. Correlation plot between individuals' metadata and microbiota composition at the family-level, (A) healthy controls, (B) NAFLD, and (C) NASH patients.
